# A descriptive study of culture media in Brazilian assisted reproduction clinics

**DOI:** 10.5935/1518-0557.20160025

**Published:** 2016

**Authors:** Ana Bartmann, Amanda Turato Barbosa do Amaral, Letícia Gonçalves

**Affiliations:** 1 Assisted Reproduction Center - Department Gynecology and Obstetrics - Medical School - University of Ribeirão Preto (UNAERP), Ribeirão Preto/SP - Brazil; 2 Human Reproduction Center of the Ana Bartmann Clinic - Ribeirão Preto/SP - Brazil; 3 Universidade Federal de Alfenas/MG - Brazil

**Keywords:** Culture media, Embryo transfer, Brazilian clinics

## Abstract

**Objective:**

The present study aimed to draw a profile of the most commonly used media and protocol characteristics from assisted reproduction technology (ART) facilities in Brazil.

**Methods:**

To obtain an overview of ART methods and culture media, a questionnaire was given to embryologists from ART clinics in Brazil. Further research in scientific papers and journals was carried out for describing the processes around Brazil, USA and Europe.

**Results:**

From the questionnaire, we found that the embryo medium mostly used is CSCM^TM^ from Irvine Scientific, represented 37.04% in Brazilian ART clinics; interestingly, 70.37% of clinics exchange the embryo media bath; however, 70.37% do not change the media type. Transfers in Brazilian clinics were variable, but day 3 transfer was a procedure seen in 37.04%. The remaining embryos are habitually maintained in prolonged cultivation in 51.85% of the clinics interviewed.

**Conclusion:**

Although there are numerous studies trying to better understand embryo culture media influences, there is a lack of evidence for choosing one as the most appropriate. In short, it is a random decision for such an essential stage of In Vitro Fertilization.

## INTRODUCTION

Infertility is an important public health issue affecting lives worldwide. Since 1978, when the first IVF baby was born, over 5 million infants have resulted from assisted reproduction technology (ART) (ESHRE, 2014). Multiple steps are taken in an In Vitro Fertilization (IVF) treatment cycle; including ovarian stimulation, oocyte retrieval, fertilization in a liquid medium, embryo selection and embryo transfer into the uterine environment (Sicignano *et al*., 2010). Understanding not only protocols and techniques, but also the products used for IVF is essential for a successful ART course. In short, the fertilization media are crucial for thriving IVF processes.

There are ART regulations worldwide as well as several federal laws in Europe, USA and Brazil. In these three places, there are regulations regarding the ART data from each clinic. Following this information governed by laws, some differences between IVF processes are easily seen. The number of clinics and egg transfer quantity are good examples. The reported clinics in Europe were 1,314 in 33 countries, in 2011(European IVF-Monitoring Consortium *et al*., 2016); in USA, there were 467 clinics in 2013 (Centers for Disease Control and Prevention *et al.*, 2015); and in Brazil, 106 by regulated data, however, a total of 130 clinics was estimated for 2014 (ANVISA, 2015). The number of egg transfers in Europe was 367,171 (European IVF-Monitoring Consortium *et al*., 2016); 73,571 in USA (Centers for Disease Control and Prevention, 2015); and in Brazil, 60,668 (ANVISA, 2015).

Despite these dissimilar numbers, advances in IVF during the last decades have been rapid and impressive; nevertheless, culture media is estimated to play a major role in this success (Chronopoulou & Harper, 2015). Beyond the types of media, the differences can be seen in protocols for preparing and using each specific medium.

A scientific and educational institution brings together more than 90% of the ART centers in Latin America (REDLARA, 2016) named RED - Latin American Register of Assisted Reproduction, published the "Manual of Procedures - Assisted Reproduction Laboratory" in 2006. This manual reveals that some ART centers have preferred to prepare media in their own laboratory, in spite of knowing the media simplicity prepared in laboratory and the importance of its quality for embryo development (REDLARA, 2006).

There are many studies offering comparisons between methods of cultivation (Marianowski *et al*., 2007), which analyses open culture and closed culture or that compare media with gene expression (Kleijkers *et al*., 2015). Therefore, the descriptive tone of this paper justified as a means to complement this field of study.

Although many studies have been carried out about embryo culture media, a recent review (Youssef *et al*., 2015) concluded for the lack of evidence to support or refute the use of any specific culture medium.

## MATERIAL AND METHODS

### a. Study design and settings

This study involved a cross-sectional evaluation of the most used IVF media in ART clinics in Brazil, discussing data from papers and the latest governmental databases from USA and Europe through searches in the NHS and Google Scholar, published between 1978 and January 2016. In addition, we collected data from Scielo and Brazilian government databases. The search for papers was done using keywords such as "cultivation media", "embryo cultivation media", "IVF cultivation media", "embryos cultivation", "culture media" or "culture media for embryos".

### b. Sampling and eligibility

A contact list was created within 55 randomized ART clinics in Brazil. To obtain as much information as possible, we contacted only the clinics working with ART. Embryologists from those clinics were contacted via e-mail or phone. In such case, embryologists answered a simple questionnaire in January 2016, and the clinics were enrolled into the study. Those who were not willing to be contacted and those who did not respond were excluded from the contact list.

### c. Recruitment, enrolment and instrumentation

An initial contact letter was mass-e-mailed to ART working embryologists on the contact list. The first contact aimed to discuss the study and the embryologists were asked to complete and e-mail the questionnaire enclosed. At the closing date of the survey, 27 (49%) embryologists had not replied to the initial letter, and 28 (51%) refused to participate or did not answer the questionnaire, leaving 27 (49%) to participate in the research.

The survey instrument was entirely focused on assessing embryologists working in ART clinics, with simple questions [Fig 1], but sufficient to elucidate the different characteristics in protocols. A 5-items self-administered questionnaire was used to collect data on: the culture media used in the services; bath exchanging and media changing - for sequential media; transfer days usually used; remaining embryos destination, and if cryopreserved or kept in prolonged cultivation.

**Figure 1:** Questionnaire given to Brazilian embryologists.

What is the culture media used in your service?
Is there bath exchanging during the process?
Is there media changing?
On which day is embryo transfer done?
What is done with remaining embryos

In Brazil, the culture media available are from: Irvine Scientific^TM^, Cook^TM^, Vitrolife, LifeGlobal, Ingámed, Origio and Sage. Here we shall focus exclusively on the media that appeared as an answer in the questionnaire.

Concerning the exchanging of bath, we aimed to know if the media culture was renewed, meaning the total removal of the first culture media without changing the bath type. For the question of changes in media, the aim was to know if the media type was changed to another brand or even to a different type from the same brand. In relation to transferring day and supernumerary embryos, the objective was to describe the different protocols followed by clinics in Brazil. The nomenclature of days was done in relation to the oocyte retrieval period. In this case, D0 representing the day of retrieval and assessment of the oocyte, D1 the fertilization day and zygote observation, D2 and D3 for the cleavage and embryo stage evaluation, D4 to D6 for the day of assessing morulae and blastocyst (Alpha Scientists in Reproductive & ESHRE Special Interest Group of Embryology, 2011).

### d. Media profiles

The media reported in the questionnaire were the following: Cleavage and Blastocyst from Cook Culture^TM^, GV blast from Ingámed^TM^, Continuous Single Culture^TM^ (CSCM^TM^) and Continuous Single Culture-Complete^TM^ (CSCM-C^TM^) from Irvine Scientific^TM^ and Global ^TM^ from LifeGlobal. Since some embryologists answered just Vitrolife without specifying the media type, there are compound specifications [Figure 2] for those four media from this brand available in Brazil, which are G-1^TM^, G-1^TM^ PLUS, G-2^TM^ and G-2^TM^ PLUS.

**Figure2:** Que Chemical characteristics of the embryo cultivation media most used in Brazil, by brand, medium type and chemical class.

		Cook Culture^TM^		Ingámed^TM^	Irvine Scientific		Life Global	Vitrolife			
		Cleavage	Blastocyst	GV blast	CSCM-C^TM^	CSCM^TM^	Global^TM^	Gl^TM^	Gl^TM^ plus	G2^TM^	G2^TM^ plus
Amino Acids	Alanine				x	x	x	x	x	x	x
	Arginine				x	x	x	x	x	x	x
	Asparagine				x	x	x	x	x	x	x
	Aspartic Acid				x	x	x	x	x	x	x
	Cystine				x	x	x	x	x	x	x
	Glutamic Acid				x	x	x	x	x	x	x
	Glutamine (stabilized)		x				x	x	x		
	Glycine	x	x		x	x	x	x	x	x	x
	Histidine				x	x	x			x	x
	Isoleucine				x	x	x			x	x
	L-alanine	x	x								
	L-arginine	x	x								
	L-asparagine										
	paragine monohydrate	x	x								
	L-asparticacid	x	x								
	L-cystine	x	x								
	Leucine				x	x	x			x	x
	L-glutamic acid	x	x								
	L-histidine		x								
	L-isoleucine	x	x								
	L-leucine	x	x								
	L-lysine	x	x								
	L-methionine	x	x								
	L-phenylalanine	x	x								
	L-proline	x	x								
	L-serine	x	x								
	L-threonine	x	x								
	L-tryptophan	x	x								
	L-tyrosine	x	x								
	L-valine	x	x								
	Lysine				x	x	x			x	x
	Methionine				x	x	x			x	x
	Phenylalanine						x			x	x
	Proline						x	x	x	x	x
	Serine						x	x	x	x	x
	Threonine						x			x	x
	Tryptophan						x			x	x
	Tyrosine						x	x	x	x	x
	Valine						x			x	x
Dipeptide	Alanyl-glutamine				x	x					
	Glycil-Glutamine						x				
Antibiotics	Gentamicin	x	x	x	x	x	x	x	x	x	x
Antioxidants	EDTA	x			x	x	x				
	Sodium citrate				x	x					
Buffer	NaHC03	x	x	x	x	x	x	x	x	x	x
Energy Substrates	D-glucose	x	x								
	Glucose			x	x	x	x				
	Hyaluronan							x	x	x	x
	Sodium Lactate			x			x				
	Sodium L-lactate				x	x					
	Sodium Pyruvate	x	x	x	x	x	x				
pH indicator	Phenol Red				x	x	x				
Protein	HSA	x	x		x				x		x
Salt and Ions	Calcium Chloride			x	x	x	x				
	Calcium Lactate	x	x								
	Calcium Pantothenate	x	x								
	Magnesium Chloride	x	x								
	Magnesium Sulphate	x	x	x	x	x	x				
	Potassium Chloride	x	x	x	x	x	x				
	Potassium Phosphate	x	x	x	x	x	x				
	Sodium Chloride	x	x	x	x	x	x				

Legend: CSCM-C^TM^: Continuous Single Culture Complete with gentamicin with HSA; CSCM^TM^: Continuous Single Sulture with gentamicin; HSA: Human Serum Albumin Serum Albumin.

The embryo cultivation media were separated in terms of brands and broken down according to their chemical characteristics, which is presented on table 2. Essential and nonessential amino acids are present in Blastocyst and Cleavage Medium from Cook^TM^, CSCM^TM^ and CSCM-C^TM^ from Irvine Scientific, Global^TM^ from Life Global and G2^TM^ and G2^TM^ Plus from Vitrolife. The culture media G1^TM^ and G1^TM^ plus from Vitrolife bear only nonessential amino acids. Only three media have dipeptides in their composition, they are: CSCM^TM^ and CSCM-C^TM^ from Irvine Scientific and Global^TM^ from LifeGlobal. The antibiotic is the same for all culture media: gentamicin. Antioxidants are present just in the Cleavage Medium from Cook^TM^, in both media from Irvine Scientific and in Global^TM^ from LifeGlobal. All of the media reported in the questionnaire have sodium bicarbonate as a buffer. As far as energy substrates are concerned, only Vitrolife had just one type (hyaluronan); in short, more types of energy supplies can be found in other media (e.g. D-glucose, glucose, Sodium lactate, Sodium L-lactate, Sodium Pyruvate). The pH is indicated by phenol red only in the media from Irvine Scientific and LifeGlobal. Human serum albumin (HSA) is already supplemented in media from Cook^TM^, CSCM-C^TM^ from Irvine Scientific and in G1^TM^ plus and G2^TM^ plus from Vitrolife. It is important to remember that products containing human originated protein in their compositions have the potential presence of contaminants sourced from such obscure nature protein (Morbeck *et al.,* 2014). The other brands have formulations with or without protein. Salt and ions are present in most of them, but in case of Vitrolife this information was not clear enough to be along with the others.

## RESULTS

After data collection, figure 3 was created to better show the content of each answer. In the case of utilized media, the most used was CSCM^TM^, represented by 10 clinics (37.04%) which responded the questionnaire. The second most used was media from Vitrolife, represented by 7 clinics (25.93%). Thirdly, 5 clinics (18.52%) reported the use of CSCM-C^TM^. In fourth came 2 clinics (7.40%) that were using GV Blast and other 2 clinics (7.40%) were using media from Cook. Lastly, only 1 clinic was using Global^TM^ media.

**Figure 3:** Summary of the answers to a 5-items questionnaire given to embryologists from Brazilian ART clinics about embryo culture media used.

Clinics	Culture media (Brand)	Bath exchanging (day)	Media changing	Transfer day	Remaining embryo
1	CSCM^TM^ (IS)	N	N	D3 or D5	Prolonged cultivation
2	CSCM^TM^ (IS)	Y (Dl)	N	D3	D3 Cryopreserved
3	Gl^TM^ &G2^TM^ (Vitrolife)	Y	Y	D3	Case and viability dependent
4	Gl^TM^ &G2^TM^ (Vitrolife)	Y	Y	D5	Cryopreserved
5	CSCM-C^TM^ (IS)	Y(D3)	N	D5	Prolonged cultivation
6	Cleav + Blast (Cook)	Y(D3)	Y	D5	Prolonged cultivation
7	Gl^TM^ &G2^TM^ (Vitrolife) Cleav + Blast (Cook)	N	Y	D5	D3 or D5 Cryopreserved
8	CSCM-C^TM^ (IS) GVBLAST( Ingamed)	YCSCM-C^TM^ (Dl) GVBLAST(D3)	N	D5	D6 Cryopreserved
9	CSCM-C^TM^ (IS)	Y (Dl)	N	D3 or D5	Cryopreserved
10	(Irvine)	Y(D3)	Y	D3	Prolonged cultivation
11	Gl^TM^ &G2^TM^ (Vitrolife)	Y	Y	D2, D3 or D5	Case and quality dependent
12	CSCM^TM^ (IS)	Y (Dl)	N	D2 or D3	Cryopreserved
13	GVBLAST( Ingamed)	Y(D3 and blastocyst)	N	D3	Cryopreserved
14	CSCM^TM^ (IS)	N	N	D3	Cryopreserved
15	CSCM^TM^ (IS)	N	N	D3	D3 Cryopreserved
16	Global^TM^ (LifeGlobal)	N	N	D4	Prolonged cultivation
17	CSCM^TM^ (IS)	Y (Dl)	N	D2 or D3	D3 Cryopreserved
18	CSCM-C^TM^ (IS)	Y (Dl)	N	D3 or D5	Prolonged cultivation
19	CSCM^TM^ (IS)	Y (Dl)	N	D3	Prolonged cultivation
20	CSCM^TM^ (IS)	Y (Dl)	N	D3 or D5	Prolonged cultivation
21	CSCM^TM^ (IS)	N	N	D3	D3 Cryopreserved
22	Gl^TM^ &G2^TM^ (Vitrolife)	N	Y	D5	Prolonged cultivation
23	CSCM^TM^ (IS)	Y (Dl)	N	Variable	Prolonged cultivation
24	Gl^TM^ &G2^TM^ (Vitrolife)	Y (sequential)	N	D3 or D5	Prolonged cultivation
25	CSCM-C^TM^ (IS)	N	N	D3	Prolonged cultivation
26	Gl^TM^ plus (Vitrolife)	Y	Y	D5	Prolonged cultivation
27	(IS)	Y(D3)	N	D3	Prolonged cultivation

Legend: IS: Irvine Scientific; Y: Yes; N: No; Dl: day one; D2: day two; D3: day three; D4: day four; D5: day five; D6: day six; Cleav: Cleavage; Blast: Blastocyst; CSCM-C^TM^: Continuous Single Culture Complete with gentamicin with HSA; CSCM^TM^: Continuous Single Sulture with gentamicin; HSA: Human Serum Albumin.

About the second question, 19 clinics (70.37%) answered they were used to bath exchange and 8 clinics (29.63%) were not. To some answers, the respondents added the day in which the bath was changed; D1 happened in 9 clinics (33%) and D3 in 6 clinics (22%).

In the third question, 70.37% of the responses were negative, compared to 29.63% that were positive for changing media type.

The question about the day of transferring, 37.04% of the clinics transfer on D3; 25.93% on D5; 18.52% on D3 or D5; 7.40% on D2 or D3. The remaining 11.10% were equally distributed in three different patterns: transfers occurring on D2, D3 or D5; D4; and variable depending on the case.

In the last question, the majority of clinics, represented by 51.85%, keep embryos in prolonged cultivation; others 18.52% answered that the embryos were cryopreserved, but did not specify the date; 14.81% of the clinics used to freeze the embryos on D3; 7.40% of the interviewees answered it would depend on each case. Lastly, 1 clinic (3.70%) cryopreserves embryos on D6 and another clinic (3.70%) cryopreserves on D3 or D5.

## DISCUSSION

The difference in the number of clinics and egg transfers in Europe, USA and Brazil can be explained by three main topics. First, those numbers are related to the quantity of countries included, the country level of development and characteristics such as environment and socioeconomic demographics. In second, the age of the population and cultural criteria influence family planning. Lastly and most relevant to IVF procedures, the earlier the embryo is transferred the more number of transfers can be carried out. In short, it is not just about being a developed or developing country that counts for the number of ART procedures.

An important discrepancy seen between the results from Brazilian clinics and the literature is about the embryo culture media. Data, described before in this paper, showed that the media is made in laboratory; however, none of the clinics answered the questionnaire with a single media done in their own laboratories. In short, although it has been described as a possible media handling, especially in Latin America, in Brazil it does not appear to be common. In conclusion, embryonic development and successful IVF treatment relies on embryo culture medium. This is a fact that can be proved by numerous literature papers in addition to the high cost of media from diverse companies. Even though the embryo media importance is known, there is a lack of studies comparing media and enough evidence to support or refute one specific medium.
